# Neoplastic and non-neoplastic swellings of the external genitalia in horses and donkeys: clinical, histopathological, and treatment outcomes

**DOI:** 10.3389/fvets.2025.1613601

**Published:** 2025-08-05

**Authors:** El-Sayed El-Shafaey, Mohammed Hamed, Yahia Amin, Khalid M. Alkhodair, Saad Shousha, Ahmed Aljazzar, Mahmoud G. El Sebaei, Esam Mosbah

**Affiliations:** ^1^Department of Surgery, Anesthesiology and Radiology, Faculty of Veterinary Medicine, Mansoura University, Mansoura, Dakahlia, Egypt; ^2^Department of Veterinary Surgery, Salam Veterinary Group, Buraydah, Qassim, Saudi Arabia; ^3^Department of Pathology, Faculty of Veterinary Medicine, Mansoura University, Mansoura, Dakahlia, Egypt; ^4^Department of Theriogenology, Faculty of Veterinary Medicine, Aswan University, Aswan, Egypt; ^5^Department of Anatomy, College of Veterinary Medicine, King Faisal University, Al-Ahsa, Saudi Arabia; ^6^Department of Biomedical Sciences, College of Veterinary Medicine, King Faisal University, Al-Ahsa, Saudi Arabia; ^7^Department of Pathology, College of Veterinary Medicine, King Faisal University, Al-Ahsa, Saudi Arabia; ^8^Department of Biomedical Sciences, College of Clinical Pharmacy, King Faisal University, Al-Ahsa, Saudi Arabia

**Keywords:** donkey, genitalia, horse, histopathological, surgical, swelling

## Abstract

**Aim:**

External genitalia swellings represent diagnostic and treatment challenges in equids. Thus, the present study aimed to describe the clinical, histopathological, and treatment outcomes of external genitalia swellings in horses and donkeys.

**Methods:**

Seventy-five equids (49 horses and 26 donkeys) from 6 months to 8 years were enrolled based on the clinical evidence of external genital swellings. The descriptive details, including sex, age, lesion location, and treatment outcomes, for each case were recorded and analyzed.

**Results:**

Twenty forms of external genital swellings were recorded and classified into neoplastic (*n* = 32; 42.67%) and non-neoplastic (*n* = 43; 57.33%) swellings. The non-neoplastic swellings included inflammatory (*n* = 30; 69.77%) and non-inflammatory lesions (*n* = 13; 30.23%). The neoplastic swellings were leiomyoma (4.00%), fibroma (1.33%), fibropapilloma (5.33%), squamous cell carcinoma (9.33%), sarcoid (14.67%), and melanoma (8.00%). The inflammatory forms included pythiosis (9.33%), balanitis (4.00%), posthitis (5.33%), balanoposthitis (6.67%), penile hematoma (2.67%), preputial hematoma (4.00%), orchitis (4.00%), vaginal hyperplasia (1.33%), and vulvitis (2.67%). The non-inflammatory forms involved Bartholin gland cyst (2.67%), persistent hymen (2.67%), hermaphrodite (1.33%), hydrocele (6.67%), and inguinal hernia (4.00%). According to the type of swelling, the treatment protocol was applied, even with conservative or surgical treatment. Sixty-seven cases (89.33%) of the treated animals were completely recovered. However, euthanasia was recommended for three cases (4.00%) with infiltrative neoplastic masses, and five cases (6.67%) died from unrelated causes to the operation.

**Conclusion:**

Accurate early diagnosis and assessment of the external genitalia swellings can offer veterinarians the opportunity for more precise prognosis and treatment decisions guidance for such challenging cases that affect the reproductive performance of horses and donkeys.

## 1 Introduction

Horses and donkeys play an important role in the Egyptian livestock agricultural economy, using them as mounts in agriculture, transport, and racing (1). Reproductive performance is a critical factor determining the profitability of the Equid industry. Regular clinical assessment of the reproductive status of equids, including the diagnosis and treatment of reproductive disorders, especially those related to the external genitalia, is essential to achieve optimal reproductive performance (2). The external genitalia swelling, even neoplastic or non-neoplastic is more prevalent in equids than in other species; it increases with age and represents diagnostic and treatment challenges (3, 4).

There are numerous causes for external genitalia swellings, such as trauma, infection, anatomical anomalies, and tumors. The external genitalia swellings in equids may be neoplastic and non-neoplastic swellings (3, 5). It was presented in various forms, which can cause discomfort and lead to further complications that reduce the reproductive efficiency of horses and donkeys (2, 6, 7).

Definitive diagnosis is crucial for predicting and providing appropriate therapy. Diagnosis of the external genitalia swellings in equids is generally based on clinical signs, diagnostic imaging, and gross appearance. In addition, histological examination was established to confirm the definitive diagnosis and provide an adequate basis for treatment (8). Treatment of the external genitalia swellings involves a variety of modalities, either surgical or conservative. Surgical treatment includes excision, segmental posthectomy, or phallectomy (3, 9). The conservative treatment is divided into systemic and topical therapy. The systemic treatment consisted of broad-spectrum antibiotics and anti-inflammatory drugs. However, the topical treatment included cold hydrotherapy massage, antimicrobial ointment, and the support truss of the penis and prepuce (9, 10). The external genitalia swellings represent diagnostic and treatment challenges in equids. Therefore, this study aimed to describe the clinical, histopathological, and treatment outcomes of some external genitalia swellings in horses and donkeys.

## 2 Materials and methods

### 2.1 Animals

A total number of seventy-five equids (49 horses and 26 donkeys) of both sexes (52 male and 23 female) at 6 months to eight years old (Mean ± SD: 40 ± 8) and 80–450 kg of weight (mean ± SD: 300 ± 25), were investigated. Animals were admitted to Mansoura Veterinary Teaching Hospital, Faculty of Veterinary Medicine, Mansoura University, Egypt, from August 2018 to January 2023. Animals were included in the study based on the clinical evidence of each swelling on the external genitalia. The Committee of Animal Welfare and Ethics at the Faculty of Veterinary Medicine, Mansoura University, approved the study protocol (No.VM.R.24.12.09).

### 2.2 Clinical examination

On presentation, all horses and donkeys admitted with external genitalia swellings underwent a thorough clinical genital examination in standing and recumbent positions. Subsequently, the superficial regional lymph nodes were examined to exclude neoplastic metastasis. The nature and location of the swellings were reported. Upon diagnosis of the type of swelling, the affected animal was subject to a conservative or surgical treatment protocol.

### 2.3 Conservative treatment

Twenty-six cases were subjected to conservative treatment including melanoma (2 cases), balanitis (2 cases), posthitis (3 cases), balanoposthitis (5 cases), penile hematoma (2 cases), preputial hematoma (3 cases), orchitis (3 cases), vulvitis (1 case), and hydrocele (5 cases). The conservative treatment for all cases was divided into systemic and topical therapy. The systemic treatment consisted of broad-spectrum antibiotics such as Amoxicillin and Clavulanic Acid 1 mL of suspension per 20 kg body weight intramuscularly (IM) (Synulox RTU, Zoetis, UK), and anti-inflammatory drugs such as Flunixin meglumine (Flunidine, Arabco, Cairo, Egypt) at 1.1 mg kg^−1^ intravenously (IV) for five successive days. At the same time, topical treatment included cold hydrotherapy massage with a 5% povidone-iodine solution for 10 min for 3 days, and antimicrobial ointment (Mastjet Forte, MSD, Egypt) above the penis and into the preputial cavity for a few days. Moreover, application of the support truss of the penis and prepuce to decrease edema formation.

### 2.4 Surgical treatment

#### 2.4.1 Preoperative preparation and anesthesia

**Figure 1:**
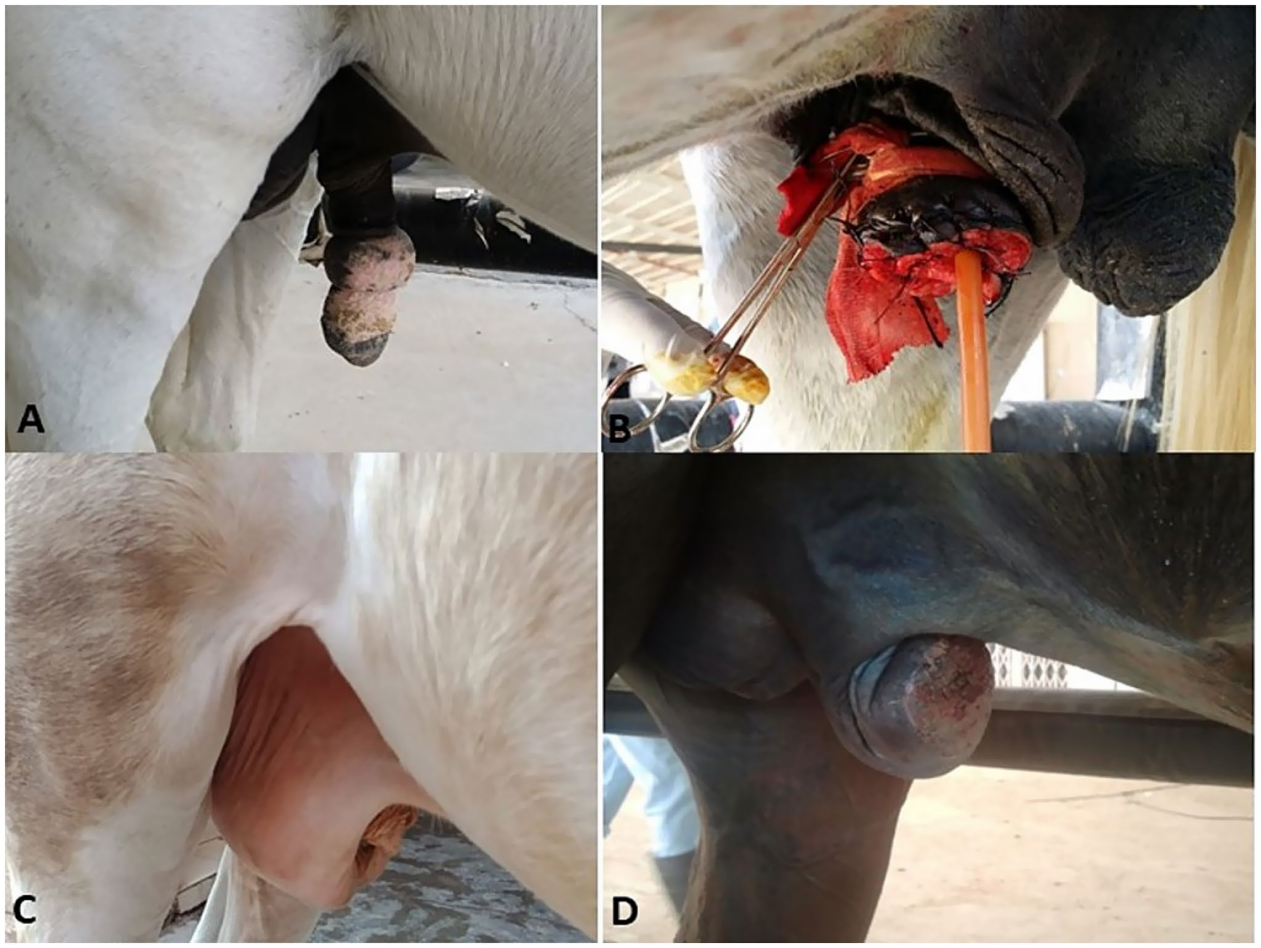
Chronic fibrous balanitis in a stallion **(A)** treated with partial phallectomy **(B)**. Posthitis **(C)** and balanoposthitis **(D)** in stallions.

**Figure 2:**
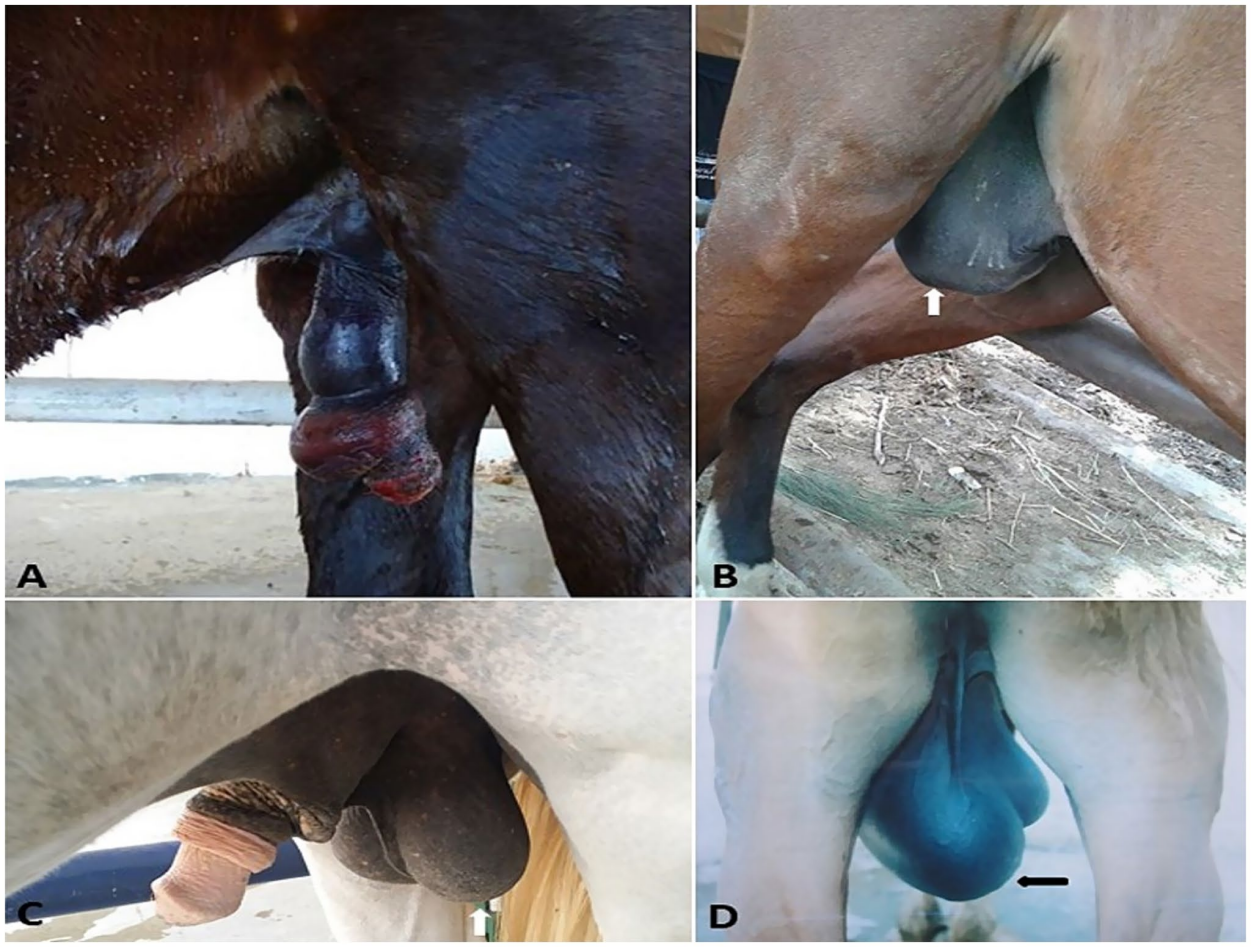
Penile hematoma **(A)**, hydrocele (white arrow, **B**), orchitis **(C)**, and inguinal hernia (black arrow, **D**) in stallions.

A complete hematobiochemical examination was performed for the animals that underwent surgical intervention; all the values were within the reference range, allowing the patient to undergo surgery. Feed was withheld for 12 h before surgery. Preoperative antibiotic cefotaxim (Cefotax, Eipico, Cairo, Egypt) at a dose rate of 3 mg kg^−1^ and flunixin meglumine (Flunidine, Arabco, Cairo, Egypt) at 1.1 mg kg^−1^ were administered IV to each animal. Sedation was induced via IV injection of xylazine HCl (Xylaject, Adwia Co., Cairo, Egypt) at 0.5 mg kg^−1^. Then, the animals were regionally or generally anesthetized according to the location of the swelling. General anesthesia was obtained using an infusion regimen of xylazine (500 mg/L) and thiopental Na (Novartis, Cairo, Egypt) (4 gm/L) at a rate of 2 mL/kg/h (11). While epidural anesthesia was achieved by injection of lidocaine HCl (Debocaine 2.5% - Al Debeiky Pharmaceutical Co., Egypt) 0.2 mg/kg in the 2nd intercoccygeal space (12). The area of operation was aseptically prepared for surgery. A catheter was placed in the urethra and advanced into the bladder as a guide to protect the urethra and to evacuate urine at the end of the procedure. According to the location and type of the masses, the surgical treatment included local excision, penile amputation (Figure 1) using William’s technique, and segmental posthectomy (8, 13). All incisions were closed with simple interrupted sutures using a 2/0 polygalactin 910 suture (Vicryl, Ethicon Inc., UK). In horses with multiple melanoma tumors, no surgical attempt was made to remove all lesions. Herniorrhaphy was performed in two males with recent non-incarcerated inguinal hernia (Figure 2) through the inguinal surgical approach (14).

#### 2.4.2 Postoperative care

The preoperative antibiotics and anti-inflammatory medications were continued for five successive days. A prophylactic dose of 15,000 IU antitetanic serum was IM injected. Animals were confined in a stall to rest for 1 week with the wound dressed and monitored daily until suture removal 10 days postoperatively. Visits and telephone inquiries obtained follow-up information for about 1 year postoperatively. Owners were questioned about the animal’s general health status, recurrence of the neoplastic masses, complications from the surgical procedure, and the ability of the operated animals to urinate freely.

### 2.5 Histopathological examination

Specimens were collected from the edges and center of the excised masses, immediately fixed in neutral buffered formalin 10%, routinely processed, and embedded in paraffin following standard histological procedures. The specimens were sectioned at 5-μm thickness and stained with hematoxylin and eosin (H&E), Van Gieson’s (VVG) for fibropapilloma, and Gomori methenamine silver (GMS) stain for pythiosis.

### 2.6 Statistical analysis

The SPSS 20 software was used for statistical analysis of the data. Normally distributed measurement data were presented as mean ± standard Error (SE) and analyzed using t-tests. Count data were presented as percentages and analyzed using the chi-square test. A chi-squared test was performed to determine the correlation between the swelling lesions, sex, and species. Pearson’s correlation analysis was used to explore the relationship between the swellings and the animal’s age. The significance level was set at *p* ≤ 0.05.

## 3 Results

### 3.1 Clinical findings

**Figure 3:**
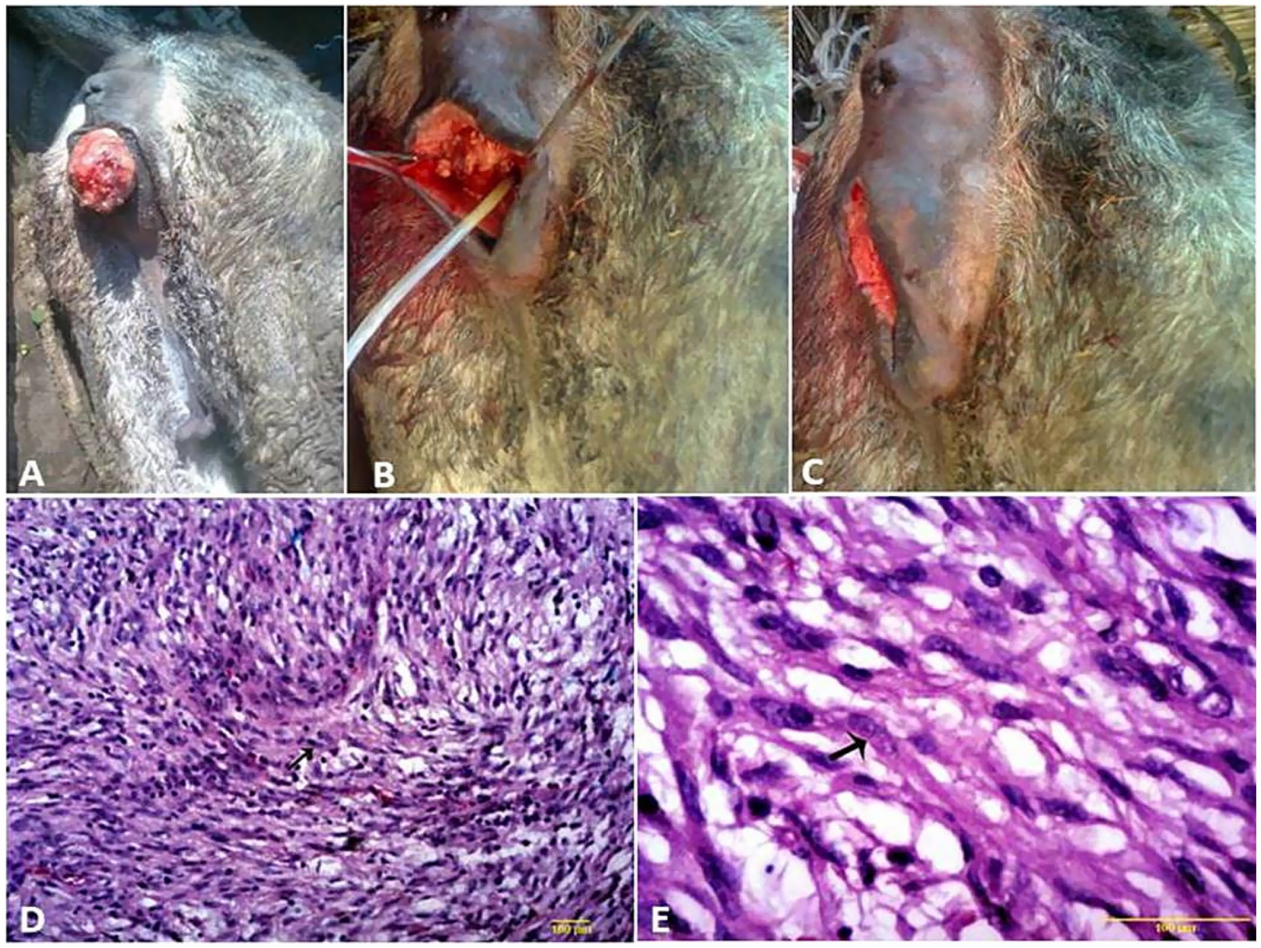
**(A–C)** Surgical treatment of vaginal leiomyoma in a she-donkey. The histological micrograph of vaginal leiomyoma revealed well-differentiated smooth muscle cells arranged in haphazardly oriented bundles (**D**, arrow) (H&E, 100 μm). Polyhedral cells with vacuolated cytoplasm and round nuclei, separated by thin fibrovascular stroma (**E**, arrow) (H&E, 200 μm).

**Figure 4:**
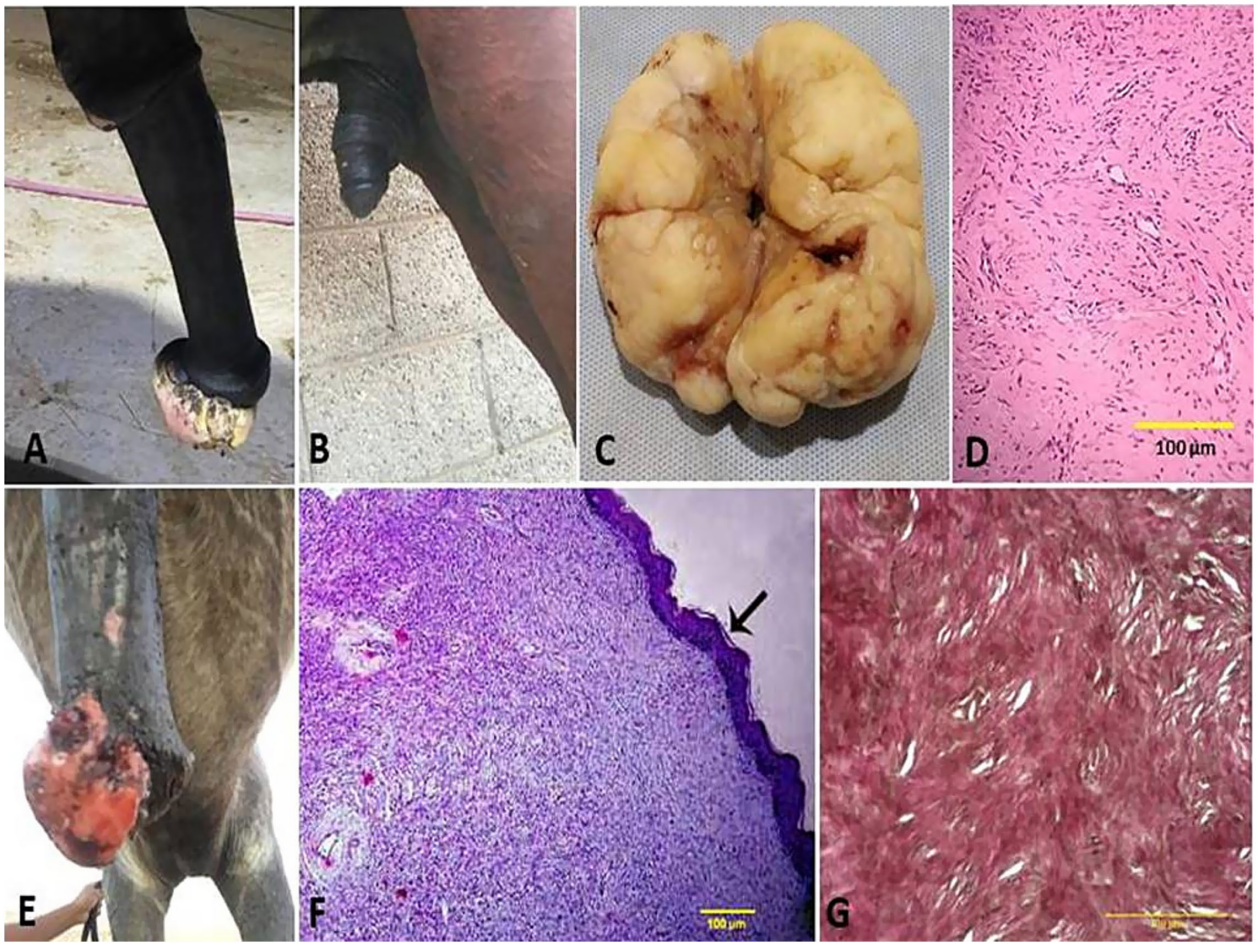
Penile fibroma on the glans penis of a stallion **(A)** and its surgical excision from the penis **(B)**. **(C)** Gross surface of the penile fibroma showed a whitish, firm, large, fibrous polypoid mass with a wet, bleeding surface. **(D)** Histological micrograph of the fibroma cells showed a capsulated nodule with whirls of fibrous connective tissue and abundant collagen fibers in between (H&E, 100 μm). **(E)** Fibropapilloma on the glans penis of a stallion. **(F)** Histological micrograph of the fibropapilloma revealed abundant loosely arranged fibrovascular stroma (H&E, 100 μm, arrow) with low to moderate numbers of spindled to stellate fibrocytes **(G)** admixed with low numbers of lymphocytes and plasma cells (VGS, 100 μm).

**Figure 5:**
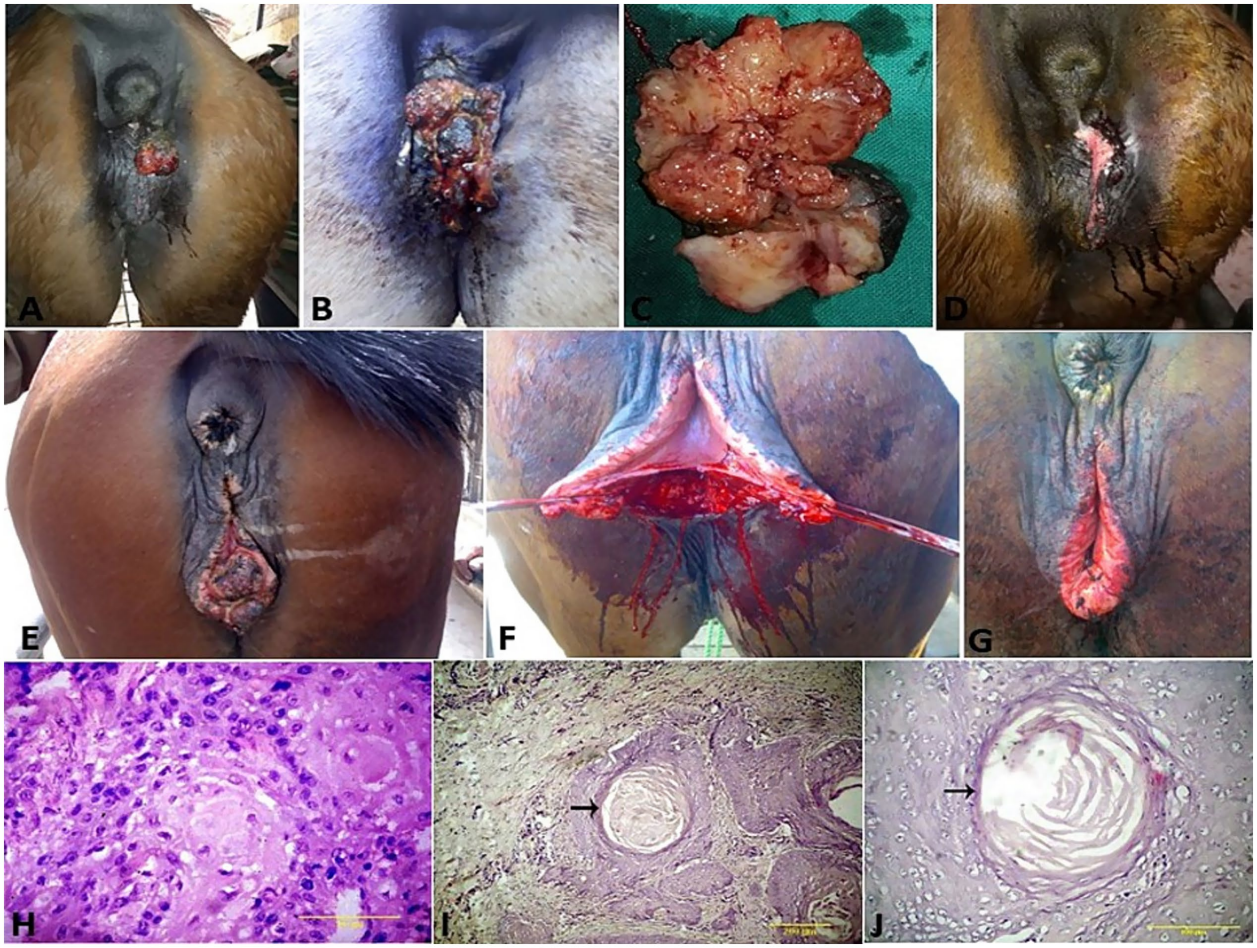
**(A–G)** SCC of the vulva and clitoris of mares and its surgical treatment. **(H)** Histological micrograph of the vulvar SCC revealed big and polygonal neoplastic cells, with large eosinophilic cytoplasm, round to oval nuclei, loose chromatin, and evident nucleoli (H&E, 100 μm). **(I,J)** SCC cell nests showing keratin formation, horn pearls, mitoses, and cellular atypia (H&E, 200 μm, arrow).

**Figure 6:**
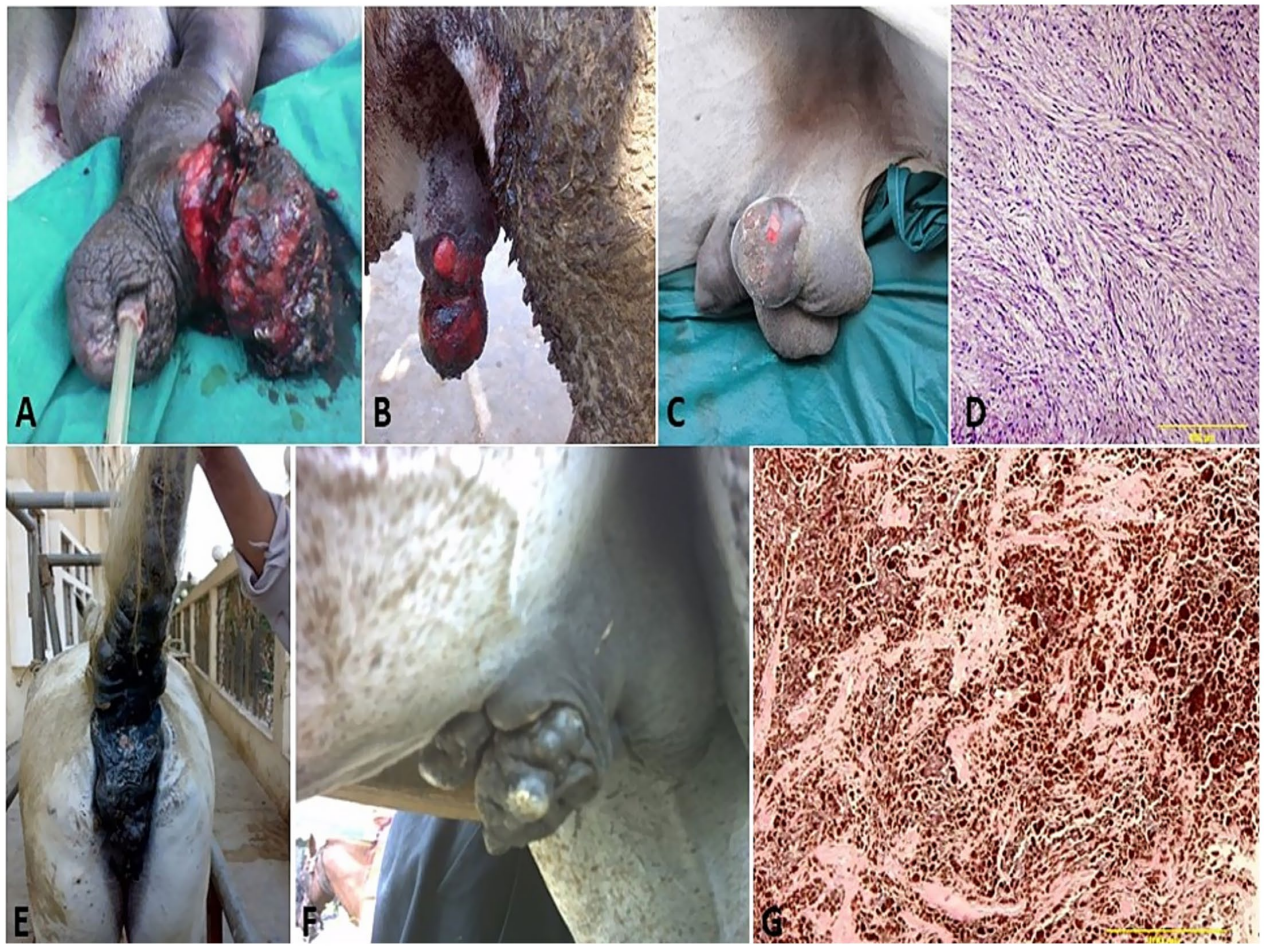
Penile **(A)**, preputial **(B)**, and scrotal **(C)** sarcoids of donkey that grossly appeared as singular or multiple cauliflower-like fibroblastic growths. **(D)** Histological micrograph of the sarcoid revealed a classical streaming and interlacing spindle-shaped fibroblast population, with massive eosinophilic infiltration (H&E, 100 μm). **(E)** Diffuse melanoma of the vulva, perineum, and tail in a whitish-gray mare. **(F)** Melanoma of the penis and prepuce in a whitish-gray stallion. **(G)** Histological micrograph revealed short interwoven bundles and closely packed nests of pigmented melanocytes interspersed with coarsely granular melanophages (H&E, 100 μm).

**Figure 7:**
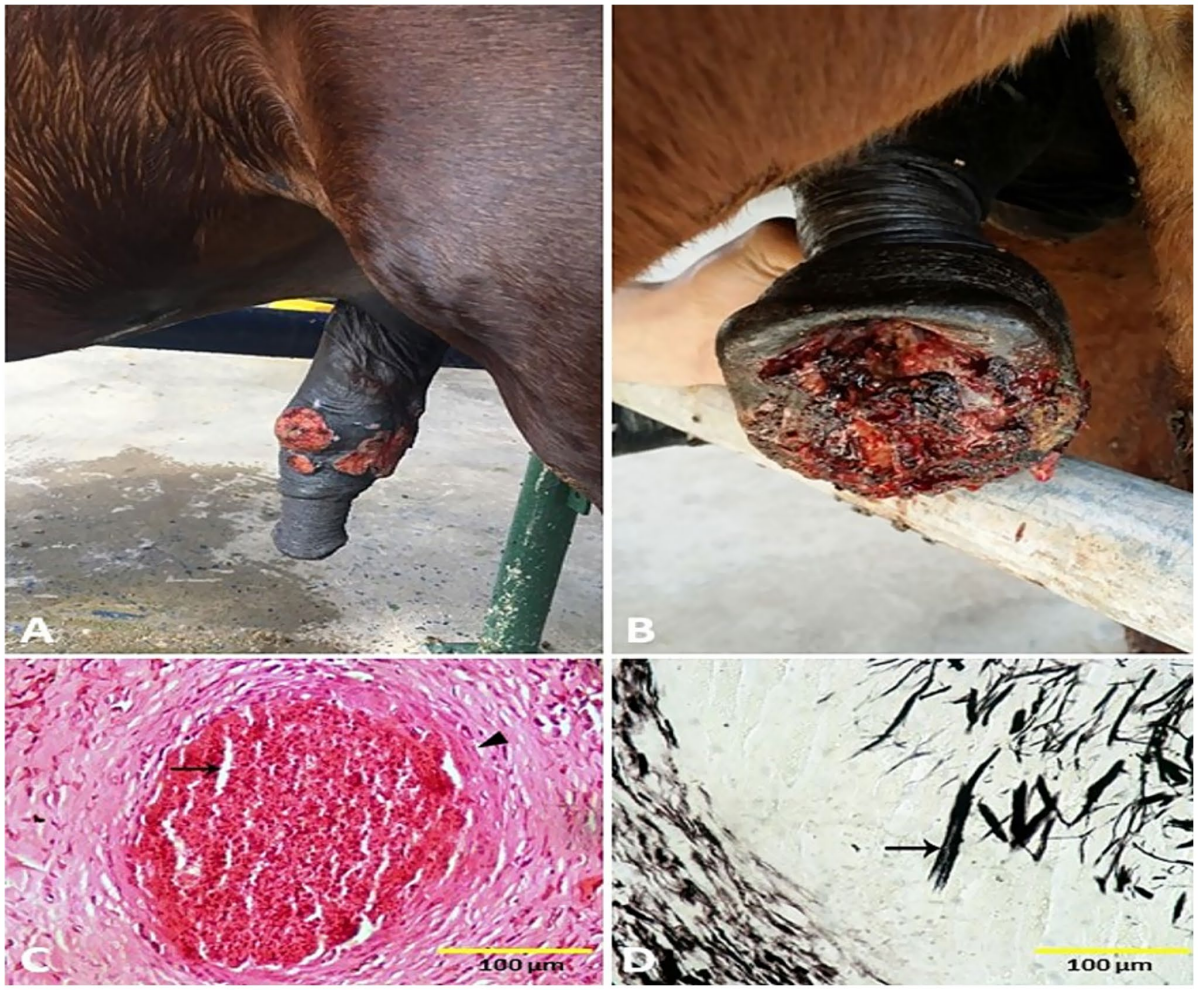
Penile **(A)** and preputial **(B)** pythiosis in stallions. Histological micrograph of pythiosis sections revealed fragments of broad hyphae surrounded by well-developed fibro-cellular capsules (arrowhead), and connective tissue rich in collagen fibers. The fibroblasts are surrounded by radiating, eosinophilic material, creating the histological appearance of the Splendore-Hoeppli phenomenon (arrow, **C**) (H&E, 100 μm). The hyphae were more easily identified by using GMS stain, where the hyphae stained dark brown to black (arrow, **D**) (GMS, 100 μm).

**Figure 8:**
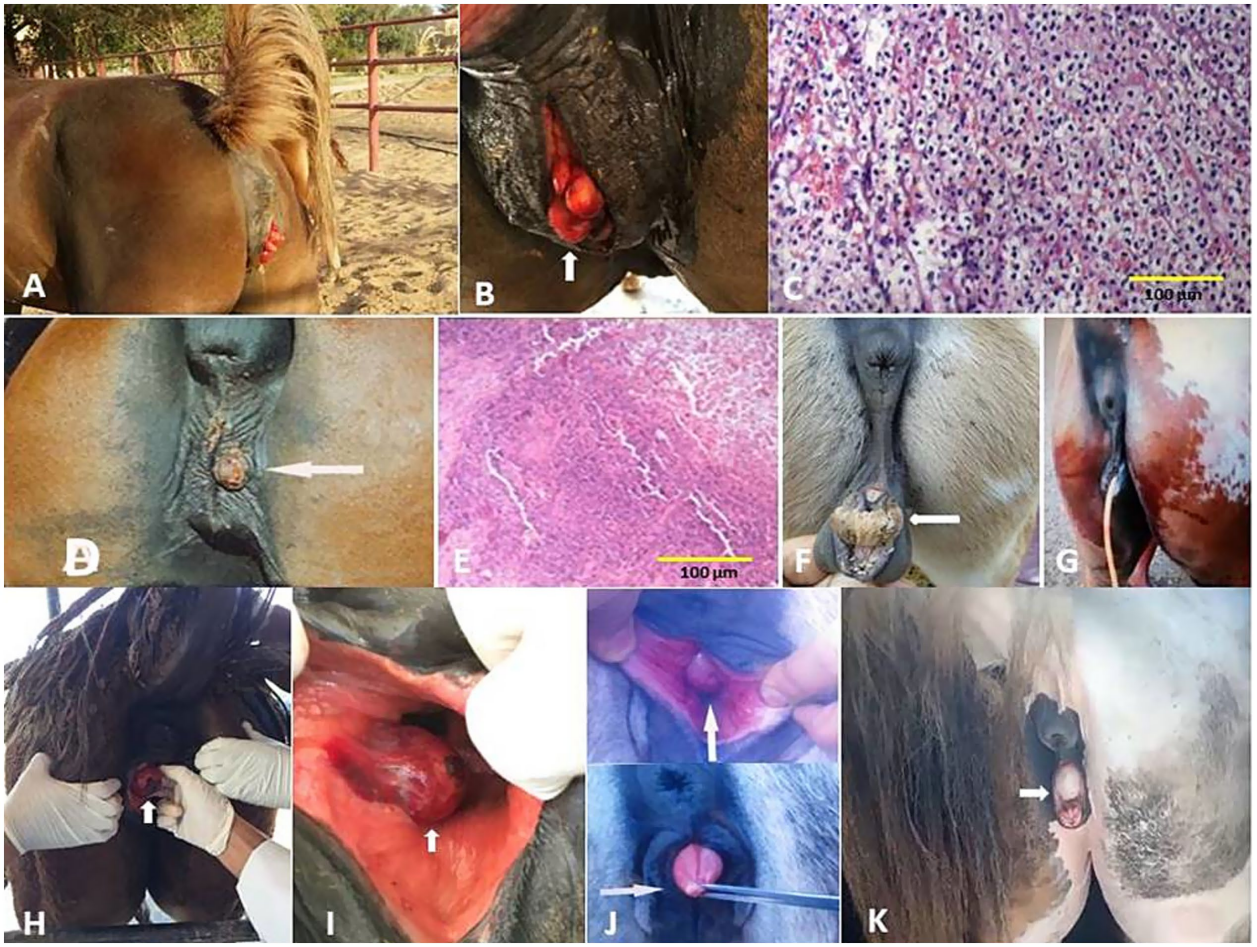
**(A)** Vaginal hyperplasia in a mare (arrow, **B**). **(C)** Histological micrograph of the vaginal hyperplasia revealed a focal area of basal cell hyperplasia, intracellular edema, and spongiosis in the vaginal epithelium with the presence of intra-epidermal vesicles and neutrophilic infiltration (H&E, 100 μm). **(D)** Infectious vulvitis in a mare showed a shallow, encrusted vulvar ulceration (arrow). **(E)** Histological micrograph of vulvitis revealed multinucleated giant cells (polykaryocytes) containing up to 20 nuclei (H&E, 100 μm). **(F)** Hermaphrodite mare (arrow) and its surgical treatment **(G)**. **(H,I)** Bartholin gland cyst in a mare (arrow). **(J)** Persistent hymen in a she-donkey (arrow) and mare (arrow, **K**).

In the present study, 75 equids (49 horses and 26 donkeys) showed various types of external genitalia swellings. The external genital swellings were classified into neoplastic swellings (*n* = 32) and non-neoplastic swellings (*n* = 43), which include inflammatory (30/43) and non-inflammatory lesions (13/43). There was a significant increase in the prevalence rate of neoplastic (42.67%) and inflammatory (40.00%) lesions compared to non-inflammatory lesions (17.33%) (Figures 1–8). The most prevalent neoplastic lesion was sarcoid, making up 14.66% of the cases. The most prevalent non-neoplastic inflammatory lesion was pythiosis, making up 9.34% of the cases. However, the most prevalent non-neoplastic non-inflammatory lesion was hydrocele (Figure 2), making up 6.67% of the cases. Based on chi-square results, there were significant correlations between the prevalence of neoplastic lesions and the sex of the affected equid (*p* = 0.009). In contrast, there was no correlation between sex and non-neoplastic, neither inflammatory nor non-inflammatory lesions. Males had a higher prevalence rate (69.33%) compared to females (30.67%). Some lesions are sex-specific, such as leiomyoma, which was recorded in females only (4.00%). While fibroma and fibropapilloma were recorded in males only (1.33%) and (5.33%), respectively. Squamous cell carcinoma (SCC) (6.67%) and melanoma (5.33%) were recorded to have a significant increase in females compared to males by 2.67% in both, respectively. At the same time, sarcoids were recorded to have a significant increase in males by 10.67% compared to females by 4.00%. In addition, there were significant correlations were demonstrated between the external genital organ and the type of lesion. The vulva showed a significant increase (23/75; 30.67%) in the lesion compared to the other external genitalia in both sexes. While the prepuce showed a significant increase (20/52; 38.46%) in the number of lesions involved in males. Moreover, there was a significant correlation between the prevalence of neoplastic lesions and the breed of the affected equid (*p* = 0.005). SCC was not observed in donkeys, and melanoma was recorded only in one donkey case. In addition, sarcoids accounted for an even higher percentage (63.70%) in the donkeys compared with the horses (36.30%). The majority of swelling lesions are predominantly observed in the age < 6 to 8 years, compared to younger ages. Sarcoid and melanoma are the two cases of neoplastic swellings that have a significant positive correlation with advancement in age (*p* ≤ 0.05), especially in ages of < 6 to 8 years. Descriptive details of external genitalia swellings in all affected animals were presented in Tables 1–3.

**Table 1:** Prevalence rate (%) of various swellings in the external genitalia of horses and donkeys.

	N. of cases (*n* = 75)	Total prevalence rate	Prevalence rate in horses	Prevalence rate in donkeys
**Neoplastic lesions (*n*=32)**				
Leiomyoma	3	4.00	1.33	2.67
Fibroma	1	1.33	1.33	–
Fibropapilloma	4	5.33	4	1.33 ^*^
SCC	7	9.33	9.33	–
Sarcoid	11	14.67	5.33	9.34 ^*^
Melanoma	6	8.00	6.67	1.33 ^*^
**Non-neoplastic inflammatory lesions (*n*=30)**				
Pythiosis	7	9.33	6.67	2.66 ^*^
Balanitis	3	4.00	1.33	2.67
Posthitis	4	5.33	4	1.33 ^*^
Balanoposthitis	5	6.67	4	2.67 ^*^
Penile hematoma	2	2.67	1.34	1.33
Preputial hematoma	3	4.00	2.67	1.33
Orchitis	3	4.00	2.67	1.33
Vaginal hyperplasia	1	1.33	1.33	–
Vulvitis	2	2.67	1.34	1.33
**Non-neoplastic non-inflammatory lesions (*n*=13)**				
Bartholin gland cyst	2	2.67	2.67	–
Persistent hymen	2	2.67	1.34	1.33
Hermaphrodite	1	1.33	1.33	–
Hydrocele	5	6.67	4	2.67 ^*^
Inguinal hernia	3	4.00	2.67	1.33

Values within rows with superscript (*) differ significantly (*p*< 0.05).

**Table 2:** Prevalence rate (%) of various swellings in the external genitalia of horses and donkeys according to sex.

	N. of cases (*n* = 75)	Total prevalence rate	Prevalence rate in males	Prevalence rate in females
**Neoplastic lesions (*n*=32)**				
Leiomyoma	3	4.00	–	4.00
Fibroma	1	1.33	1.33	–
Fibropapilloma	4	5.33	5.33	–
SCC	7	9.33	2.66	6.67 ^*^
Sarcoid	11	14.67	10.67	4.00 ^*^
Melanoma	6	8.00	2.67	5.33 ^*^
**Non-neoplastic inflammatory lesions (*n*=30)**				
Pythiosis	7	9.33	9.33	–
Balanitis	3	4.00	4.00	–
Posthitis	4	5.33	5.33	–
Balanoposthitis	5	6.67	6.67	–
Penile hematoma	2	2.67	2.67	–
Preputial hematoma	3	4.00	4.00	–
Orchitis	3	4.00	2.67	–
Vaginal hyperplasia	1	1.33	–	1.33
Vulvitis	2	2.67	–	2.67
**Non-neoplastic non-inflammatory lesions (*n*=13)**				
Bartholin gland cyst	2	2.67	–	2.67
Persistent hymen	2	2.67	–	2.67
Hermaphrodite	1	1.33	–	1.33
Hydrocele	5	6.67	6.67	–-
Inguinal hernia	3	4.00	4.00	–-

Values within rows with superscript (*) differ significantly (*p*< 0.05).

**Table 3:** Correlation between age and various neoplastic swellings in the external genitalia of horses and donkeys.

**Neoplastic swellings**	**Pearson’s Correlation**	***P value***
Leiomyoma	0.44	0.55
Fibroma	0.25	0.74
Fibropapilloma	0.77	0.22
SCC	0.77	0.22
Sarcoid	0.94	0.05
Melanoma	0.91	0.08

Correlation is significant when the *p*-value is ≤ 0.05.

### 3.2 Treatment outcomes

Sixty-one cases (81.40%) of the 75 external genitalia swellings were discharged from the clinic without any complications. In addition, six cases (8.00%) were discharged after treatment of postoperative complications such as intermittent hemorrhage, edema, balanitis, posthitis, and recurrence. Euthanasia was recommended for three cases (4.00%) with infiltrative neoplastic masses (one case of SCC and two cases of melanoma) that did not respond to treatment and showed aggressive recurrence along a wide area, with deterioration of the general health condition. Euthanasia was performed using an overdose of thiopental Na (50–200 mg/kg) quickly administered intravenously as a bolus. However, five cases (6.60%) died, four of them postoperatively (2 cases of SCC, melanoma, and pythiosis) from unrelated causes to the operation, and the 5th one (balanoposthitis) was after prolonged conservative treatment without any response.

### 3.3 Histopathological findings

Vaginal leiomyoma appeared grossly as a discrete, pink, greasy firm multilobular encapsulated mass approximately 25 cm in diameter protruding from the vulva wall. On the cut section, the tumor was whitish and opaque with a smooth appearance (Figures 3A–C). Histopathological evaluation of the tumor revealed densely packed spindle-shaped cells arranged in broadly woven fascicles and bundles with eosinophilic fibrillar cytoplasm and a large, centrally placed, cigar-shaped vesicular nucleus. The tumor cells were supported by a thin fibrovascular stroma containing numerous capillaries. The neoplastic cells were large, moderately monomorphic, and had ill-defined cell borders (Figures 3D–E). Gross examination of the penile fibroma revealed a large fibrous polypoid mass on the glans penis that was firm in consistency and whitish in color with a wet bleeding surface. Microscopically, tumor cells showed a capsulated nodule with whirls of fibrous connective tissue that run in all directions, with abundant collagen fibers in between (Figures 4A–D). The fibropapilloma grossly revealed multiple, smooth, glistening, grayish-pink, 1.0–5.0 cm diameter, exophytic, nodular masses. Microscopically, the masses consisted of abundant loosely arranged fibrovascular stroma with low to moderate numbers of spindled to stellate fibrocytes, admixed with low numbers of lymphocytes and fewer plasma cells. The overlying epithelium was mildly to moderately hyperplastic with short anastomosing rete ridges consisting of well-differentiated and well-organized epithelial cells (Figures 4E–G).

SCCs appeared as a proliferative cauliflower-like firm mass with an irregular surface and multifocal red-to-black spots (Figures 5A–G). Histopathological examination of the SCCs revealed small aggregates, irregular islands or trabeculae, and nests of neoplastic keratinocytes that proliferate downward from the epidermis and invade the sub-epithelial stroma of the dermis. Neoplastic cells were big and polygonal, with large eosinophilic cytoplasm, round to oval nuclei, loose chromatin, and evident nucleoli. (Figures 5H–J). While sarcoids appeared grossly as singular or multiple cauliflower-like fibroblastic growths (Figures 6A–C). Microscopically, there was a classical streaming and interlacing spindle cell population, and a “picket fence” appearance at the epithelial interface, and long, thin, dissecting rete ridges are typical characteristic features of equine sarcoids (Figure 6D). However, melanoma appeared smooth, firm, small, nodular, and hairless with darkly pigmented intact skin (Figures 6E,F). On histopathological examination, the tumor, characterized by a variety of cell patterns ranging from sheets to streams and nests of melanocytes, shifts the diagnosis to melanotic melanoma. Cell morphology varies from being epithelioid to spindle-shaped. Melanin pigmentation was mild, and it was seen interspersed between the tumor cells (Figure 6G).

Lesions of pythiosis grossly appeared as a cutaneous nodule that progressed to an alopecic, exudative, ulcerative, edematous, granulomatous mass. Squeezing of that mass led to the discharge of serosanguinous or bloody exudates from multiple sinus tracts. The recorded masses ranged from 5 to 20 cm in diameter and 1 to 5 cm in thickness. Transverse sections of the excised masses revealed characteristic whitish-yellow, coral-like materials named kunkers or leeches within the sinus tracts and the granulomatous tissue. Kunkers were firm and gritty in consistency, with a diameter ranging from 0.5 mm to 5 mm (Figures 7A,B). Histological examination of pythiosis sections revealed fragments of broad hyphae surrounded by well-developed fibro-cellular capsules, connective tissue rich in collagen fibers. The fibroblasts are surrounded by radiating, eosinophilic material, creating the histological appearance of the Splendore-Hoeppli phenomenon. The hyphae were more easily identified by using GMS stain, where the hyphae stained dark brown to black (Figures 7C,D). However, infectious vulvitis early lesions grossly appeared as a red vesicle or pustule on the vulva of the mare. Later, the uncomplicated lesions appeared circular and pock-like. Sequentially and after epidermal sloughing of the necrotic dome of the pustule, a shallow, raw, or encrusted erosion or ulcer appears. The size of the lesions increases up to 1–2 cm (Figure 8D). Histopathological examination of vulvitis showed multinucleated giant cells (polykaryocytes) containing up to 20 nuclei (Figure 8E).

## 4 Discussion

The external genitalia are covered with skin and can thus be affected by tumors of epithelial or mesenchymal origin. In addition, being subjected to many infectious or traumatic factors that induce swellings should be differentiated from neoplasia (15). Dermal tumors are the most common forms in equids, of which sarcoids, SCCs, and melanomas are prevalent. The causes of cellular transformation are multifactorial, including various infectious agents, trauma, inflammation, ultraviolet radiation, castration, coat color, poor genital hygiene, and lack of skin pigmentation (2, 7, 16). Equine sarcoid is the most common skin tumor in equids (5). They are nonmetastatic but frequently recur locally. These tumors do not metastasize and show variable clinical manifestations, ranging from local aggressive infiltrative growth to the less common “spontaneous” regression that can make it hard to differentiate them from SCC, proliferative granulation tissue, cutaneous habronemiasis, and other conditions (17). In this study, the high prevalence of equine sarcoid was detected among donkeys, with increased risk among males. This tumor can affect animals at any age (18). In this study, the majority of cases at tumor onset were in 6-month-old to 6-year-old animals (54.55%). These findings were in agreement with other researchers who reported a higher prevalence in younger individuals (5, 17, 18).

SCC is the most common tumor of the vulva, penis, and prepuce of the equids mainly in aged ones. It generally occurs on non-pigmented areas of the skin and mucous membranes. Metastasis can occur, making early diagnosis very important (10). The clinical manifestations and gross appearance of SCC are of great diagnostic significance in equids (2, 15). In this study, SCCs appear grossly as proliferative ulcerated lesions with poorly defined borders of varying sizes, many of which have a cauliflower-like appearance, and tend to bleed easily. In addition, the Histomicrograph of SCCs was pleomorphic, round to caudate in shape, exhibiting prominent anisokaryosis and anisocytosis. These gross and histopathological features aligned with the previous observations (19, 20).

Melanomas occur most commonly in gray-coated horses. Although they are usually benign, malignant transformation can occur (21). The importance of melanoma lies in its high prevalence, its consequences on equids health, especially the urogenital system, and its negative effect on the performance of athletic ones (22). Larger melanomas may cause physical obstruction of the anal sphincter, penis, and prepuce, or vulvar commissure, which may result in dyschezia, dysuria, and difficulty with coitus and parturition (21). Melanomas are common, generally slow-growing, locally invasive tumors, estimated to occur in approximately 80% of aging grey horses (6). Treatment of melanomas depends on the localization, the size, and the number of masses. Surgery in an early development stage is possible; however, if the tumors fuse to form a tumor tissue plate, removing it becomes very difficult because of the extensive and invasive character of the tumor tissue plate (22). All melanoma cases in the present study are aging gray Arabian horses and a donkey. This could be attributed to the higher number of equids with gray coat color in these breeds. Gray aging equids that develop melanoma usually have a poor prognosis, leading to death due to systemic spread (21). In addition, during aging, grey horses appear to develop a disturbance in the metabolism of melanin, leading to focal hyperplasia of the melanocytes and local over-production of dermal pigment, which can predispose to malignant transformation. In this study, our cases appeared firm, nodular, and hairless masses with intact skin at the penis, prepuce, and vulva. It was surgically excised in four cases, with a successful outcome and good recovery in three cases. However, two cases with greatly extensive melanoma in the perineal region were euthanized after failure to respond to any treatment trials.

Leiomyomas are benign smooth muscle neoplasias that occur in various organs, including the reproductive system. Vaginal leiomyoma can be oval or round, usually as well defined, capsulated, a single or multiple structures (23). Diagnosis of leiomyoma has to be confirmed by histopathology. The most common treatment choice is surgical intervention with extirpation of the mass through vaginotomy with or without episiotomy (2, 15). Although these tumors are most common in humans and are relatively frequent in mares and other animal species, leiomyomas in donkeys are rare (7, 24). To the best of the authors’ knowledge, this is the first report on vaginal leiomyoma in She-donkey. Little is known about the etiology and pathogenesis of leiomyoma; it may be associated with ovarian follicular cysts or estrogen-secreting tumors (23).

In this study, a case of fibroma and four cases of fibropapilloma were recorded on the body of the penis, prepuce, and scrotum. Similar findings were reported by Lee et al. (4) and Gardiner et al. (9). Differential diagnoses, including squamous papilloma, SCC, habronemiasis, balanitis, and trauma-induced granulation tissue, were considered but ruled out because of the lack of appropriate microscopic features, respectively, and the absence of any history of trauma.

In the present study, two cases of equine infectious vulvitis were recorded. These lesions were characterized by the formation of papules, vesicles, pustules, and ulcers on the vulva of mares. Similar findings were reported by Thorsteinsdóttir et al. (25) who stated that equine venereal infectious vulvitis or equine coital exanthema (ECE) caused by equid alpha herpesvirus 3 (EHV-3) is a contagious venereal disease. In addition, the virus is probably in the herpesvirus group but differs immunologically and culturally from the virus of equine rhinopneumonitis. In this study, different types of non-neoplastic external genital swellings were recorded, including pythiosis, balanoposthitis, posthitis, hematoma, orchitis, vulvitis, and vaginal hyperplasia. Differential diagnosis of such swellings is challenging, as several different types of swellings share similar clinical characteristics and may be confused clinically with other tumors. The histopathological analysis of every surgically removed tissue should be performed in order not to miss a tumorous condition, even if it does not show any clinical symptoms. Similar recommendations were reported by Arjun et al. (26), Bademkiran et al. (27), Pandey et al. (28), Chisholm et al. (29), and Pompermayer et al. (30).

Donkeys are not small horses; they have their own unique biology, which includes differing health conditions. One key way donkeys differ from horses is the types of tumors they develop (3, 7). Our study demonstrates commonalities as well as differences in neoplastic lesions of donkeys and horses. In the present study, SCC was not observed in donkeys, and melanoma was recorded only in one case, even though their coat color is also grey. This could be attributed to species differences known to be important with papillomavirus pathogenesis; donkeys are less or not susceptible to this infection, accounting at least in part for the scarcity of SCC and melanoma (5, 21). Although most donkeys are fully pigmented, there are many white-patterned donkeys, and male donkeys with no white patterning may have pink penile skin; thus, pigmentation differences alone do not account for differences in SCC occurrence between horses and donkeys. In addition, equine sarcoid is the most common tumor in both donkeys and horses, although sarcoids in the donkeys of our study accounted for an even higher percentage (63.70%) compared with the reported percentage of sarcoids in horses (36.30%). This high relative prevalence of sarcoids in donkeys could be attributed to the absence of SCC and scarcity of melanoma, both common skin tumors in horses (5, 7, 21). Thus, understanding the differences in carcinogenesis between these closely related species may prove useful to veterinary diagnosticians as well as to researchers pursuing pathogenic mechanisms of equids disease.

Sex predilections for many tumors are poorly described in equid populations. There has been no attempt to identify whether the most commonly diagnosed tumor types have changed over time (2). The risk and types of external genitalia swellings are strongly associated with the animal’s sex that affected by the anatomical variation and hormonal influences. However, certain types of tumors are more prevalent in one sex than the other, depending on the species, age, environmental, and genetic factors (7). The sex hormones as estrogen, progesterone and testosterone play a critical role in the development of some genital tumors. In this study, the associations between sex and swelling type were evaluated with evidence of significant correlations between them (*p* = 0.009). Our results revealed that females accounted 30.67% and males accounted 69.33%. Some lesions are sex-specific, such as leiomyoma, which was recorded in females only (4.00%). While fibroma (1.33%) and fibropapilloma (5.33%) were recorded in males only. SCC and melanoma were recorded to have a significant increase in females by 6.67 and 5.33% compared to males by 2.67% in both, respectively. At the same time, sarcoids were recorded to have a significant increase in males by 10.67% compared to females by 4.00%.

The presence or absence of specific external genital organ dictates the types of cancers that can develop. Understanding the type of lesion, its location, and associated symptoms is crucial for diagnosis and treatment of external genitalia lesions. The location of the lesion within the external genitalia can vary, with some areas being more prone to certain types of neoplasia. In addition, there were significant correlations were demonstrated between the external genital organ and the type of lesion. The vulva showed a significant increase (23/75; 30.67%) in the lesions compared to the other external genital organs in both sexes. While the prepuce showed a significant increase (20/52; 38.46%) in the number of lesions involved in males. This could be attributed to the anatomical situation of the vulva and prepuce that subject them to the chronic irritation and direct contact with the risk factors that induce external genital lesions.

The incidence of external genital tumors is more strongly age-dependent, especially for the malignant ones (2). In our study, 38.67% of the affected animals were between 4 years to 6 years old, and about 34.67% were between 6 years to 8 years old. In addition, results showed that the swelling affection in different ages reveals that the old age of < 4 years to 6 years and < 6 years to 8 years are more susceptible to the swelling lesion affection compared to younger ages. This could be attributed to the increase of the risk of some genital neoplasia with age in both males and females. However, other studies have reported age as a tumor risk factor but have not distinguished between neoplasms (7, 17).

Treatment of External genital swellings is dependent upon the location and severity of the lesion as well as the presence or likelihood of metastases. Options include excision alone or in combination with phallectomy, en bloc resection, or posthectomy (8). Phallectomy is indicated for horses with invasive irreparable lesions of the distal penis as a neoplastic invasion of the penile tunic of the glans, body, or urethral orifice. Phallectomy is usually performed to salvage the horse for purposes other than breeding. Hemorrhage, urethral stenosis, and preputial edema are frequently observed after phallectomy and can be managed easily (3). Various techniques of phallectomy (i.e., Vinsot’s, Williams’, and Scott’s) have been described with significance in reducing urethral stricture (13). Partial phallectomy with or without retroversion has been described in the standing horse and avoids the costs and risks involved with general anesthesia (8, 13). The technique of partial phallectomy performed in this study used the incorporation of simple interrupted and continuous sutures to compress the cavernous tissue of the corpus cavernosum penis to reduce postoperative swelling and the risk of wound dehiscence. Circumferential posthectomy is indicated for removing tumors on the loose preputial tissues without the involvement of the underlying penile tunic. However, segmental posthioplasty is shown for removing large circumferential penile or preputial lesions that do not extend beyond the dermis, while preserving the ‘telescopic function’ of the prepuce (26). In the present study, we performed it with conscious and standing animals, contained in the horse stock, with an anesthetic sedation protocol and local anesthesia, because it is a minimally invasive surgery. Potential complications of circumferential posthectomy include edema, hematoma, infection, and dehiscence. Scott and Hughes (3) and Arjun et al. (26) reported similar findings. Extensive resection of the vulva may be necessary if a large tumor is present. Ablation of the clitoral fossa is often required when vulvar SCC involves the clitoris. If a large portion of the vulva was removed, vulvoplasty (i.e., Caslick’s procedure) may be needed to prevent pneumovagina (20). The present study performed valvuloplasty in cases of vulvar swellings, such as SCC and infectious vulvitis.

Tumor recurrence is possible after surgical excision, which can be expected in 17–25% of cases; therefore, wide surgical excision is recommended. The prognosis for complete recovery after local excision is excellent if adequate tissue margins are obtained (2, 3). In the present study, three cases of SCC, melanoma, and balanoposthitis undergo recurrence. This may be because those horses or donkeys were affected by a more advanced stage of disease at the time of surgery than was appreciated. This might have involved spread to the inguinal lymph nodes or the presence of a tumor in the tissues remaining after surgery. This was consistent with the findings of previous studies (3, 31).

There are some limitations of this study; it required further deep research on large sample to provide more data about the impact of external genitalia swellings on equid production. Investigate the related risk factors and the pathogenic mechanisms for the differences in carcinogenesis between different equid-related species. Include extra accurate ancillary diagnostics for more potent case analysis. Moreover, apply the conservative chemotherapy or radiotherapy treatments in conjunction with the surgical excision of malignant cases.

## 5 Conclusion

Accurate early diagnosis and assessment of the external genitalia swellings can offer veterinarians the opportunity for more precise prognosis and treatment decisions guidance for such challenging cases that affect the reproductive performance of horses and donkeys.

## Data Availability

The original contributions presented in the study are included in the article/supplementary material, further inquiries can be directed to the corresponding authors.
